# Clinical Utility of PROSTest: A Prospective Study Suggesting Reduction in Unnecessary MRI and Biopsy in Men Evaluated for Prostate Cancer

**DOI:** 10.3390/cancers18050871

**Published:** 2026-03-08

**Authors:** Kambiz Rahbar, Martin Bögemann, Philipp Papavasilis, Abdel Halim, Mark Kidd

**Affiliations:** 1 Department of Nuclear Medicine, University Hospital Muenster, 48149 Muenster, Germany; kambiz.rahbar@ukmuenster.de; 2 West German Cancer Centre Muenster (WTZ), 48149 Muenster, Germany; martin.boegemann@ukmuenster.de (M.B.);; 3 Department of Urology, University Hospital Muenster, 48149 Muenster, Germany; 4 Wren Laboratories, Branford, CT 06405, USA; ahalim@wrenlaboratoratories.com

**Keywords:** liquid biopsy, gene expression, PCR, prostate cancer screening, PROSTest, PSA, MRI

## Abstract

**Background/Objectives**: Early detection of prostate cancer (PCa) enables timely therapeutic intervention and improved clinical outcomes. Screening strategies are increasingly individualized and now incorporate multiparametric MRI findings, reported using the Prostate Imaging Reporting and Data System (PI-RADS), to refine biopsy decision-making. PROSTest is a novel machine learning (ML)-enhanced, 30-gene mRNA liquid biopsy assay developed to detect PCa from whole blood. In this prospective study (NCT06872619), we evaluated whether PROSTest could function as a pre-biopsy triage tool to inform biopsy decisions while preserving sensitivity for clinically significant prostate cancer (csPCa). **Methods**: Of 121 men evaluated, 111 (91.7%) completed the full diagnostic work-up—including PSA testing, PROSTest analysis, and PI-RADS assessment—and subsequently underwent image-guided biopsy. Peripheral blood samples for PROSTest were collected prior to biopsy. RNA-stabilized samples underwent RNA isolation followed by reverse transcription and quantitative PCR. Gene expression data were processed using a proprietary machine learning algorithm to generate a continuous range from 0 to 100. A clinically validated cut-off ≥ 50 was applied to produce a binary (positive/negative) result. The diagnostic accuracy of PROSTest was assessed against histology-confirmed prostate cancer. **Results**: The median age of participants was 69 years (47–83 years) and the median PSA was 7.5 ng/mL (IQR: 5.8–11.4 ng/mL); most patients (104 of 111; 93.7%) had a PI-RADS score of three to five. PCa was diagnosed in 97 men (87.4%) including eight in ISUP Grade Group (GG) 1, 46 in GG2, 33 in GG3, three in GG4 and seven in GG5. PROSTest was positive in 102/111 (91.9%). Among men with biopsy-confirmed PCa, diagnostic accuracy was 99% (93/94). Of the 17 men without histologic evidence of disease, eight (47%) were PROSTest-negative. The overall accuracy was 91% (84.1–95.6%) with an NPV of 89% (51.6–98.4%). Among the nine patients with positive PROSTest but negative biopsy, PI-RADS scores were 4 (*n* = 6), 3 (*n* = 1), and 2 (*n* = 2). **Conclusions**: PROSTest demonstrated an overall accuracy of 91% (95% CI: 84.1–95.6%) with an NPV of 89%. Among men without a detectable prostate cancer on biopsy, 47% (8/17) were PROSTest-negative. These results suggest that PROSTest may serve as a useful pre-biopsy triage assay.

## Simple Summary

This study demonstrates that PROSTest achieves high diagnostic accuracy (>90%) for risk stratification of prostate cancer (PCa) in men undergoing multiparametric MRI and image-guided biopsy. The assay effectively distinguished patients with clinically significant disease from those without histologic evidence of malignancy and was consistently negative in biopsy-negative individuals, supporting a strong negative predictive value. These findings highlight the potential utility of PROSTest as a pre-biopsy triage tool to improve patient selection, reduce unnecessary biopsies, and limit overdiagnosis. Integration of PROSTest into current diagnostic pathways may enhance precision in PCa evaluation and optimize clinical decision-making.

## 1. Introduction

Prostate cancer (PCa) remains one of the most commonly diagnosed malignancies and a leading cause of cancer-related death among men in the United States and the European Union, especially in high-risk populations such men of African genetic heritage and military veterans [1,2,3]. Early detection of PCa enables timely and potentially curative intervention and remains a cornerstone of management for this high prevalence disease. Current diagnostic pathways rely primarily on serum prostate-specific antigen (PSA) levels and digital rectal examination (DRE) as initial triage tools [4]. However, PSA lacks specificity and has limited positive predictive value (PPV), contributing to the overdiagnosis of indolent cancers and the frequent performance of unnecessary prostate biopsies. Many biopsies are either negative or identify clinically insignificant disease which may, subsequently, be overtreated [5,6].

Over the last decade, prostate MRI findings (PI-RADS) have been increasingly used to help predict the need for a prostate biopsy and thus potentially avoid unnecessary procedures as well as steering biopsies and finding a higher proportion of significant prostate cancer [7,8]. In current clinical practice, men with PSA levels ≥ 3 ng/mL or a positive DRE are frequently referred for multiparametric magnetic resonance imaging (mpMRI) as a triage step before biopsy [9,10]. However, mpMRI presents limitations in specificity, mainly because of inter-reader variability outside of specialized centers and in real-world setting [11,12].

PROSTest is a novel, blood-based, multigene expression assay that quantifies whole-blood mRNA levels of 27 prostate cancer-associated genes (Supplementary Table S1) normalized to three housekeeping genes (HKGs) using qPCR and a proprietary machine learning algorithm [13]. The assay produces a continuous risk score ranging from 0 to 100. A clinically validated cut-off of ≥50 is applied to dichotomize results into “positive” or “negative” categories for prostate cancer risk stratification. Unlike PSA and PSA-derived indices (e.g., PHI, 4K score), being performed on mRNA extracted from whole blood, PROSTest reflects the holistic image of tumor biology by capturing circulating tumor RNA, RNAs from circulating tumor cells (CTCs), exosomes, tumor-educated platelets as well as cancer-enhanced immune cells. The procedure is made feasible by Wren’s proprietary RNA stabilization buffer, which preserves RNA integrity for ≥10 days at ambient temperature [13].

This study prospectively evaluated PROSTest in real-world practice as a reflex test guiding the decision to proceed with MRI in men aged ≥45 years with elevated PSA or positive DRE, aiming to confirm its potential value in precluding unnecessary imaging and biopsy or helping to guide further follow-up while maintaining high sensitivity for prostate cancer detection (NCT06872619).

## 2. Materials and Methods

This prospective, single-center study was designed to pose no risk to enrolled patients. All subjects were managed *per* current guidelines and institutional practice. PROSTest was not used for making medical decisions.

Subjects: Men aged ≥45 years with prescreened PSA levels ≥ 3.0 ng/mL or a positive DRE, potentially at risk for prostate cancer, were subjected to an mpMRI. We specifically excluded any patient diagnosed with prostate cancer or with a history of the disease. Eligibility criteria were based on recommendations for screening (EAU-EANM-ESTRO-ESUR-ISUP-ISOG guidelines) [14]. Blood samples for PSA and PROSTest measurements were collected prior to MRI and biopsy. The study was performed in accordance with the Standards for Reporting of Diagnostic Accuracy Studies (STARD) guidelines [15]. Ethical approval was obtained from the Institutional Ethics Committee of the Medical University of Munster (2007-467-fS) and the PROSRegistry (NCT06872619). Written informed consent was obtained from all participants prior to study enrollment. This clinical study focuses on assessing the utility of molecular-based blood tests, e.g., PROSTest both as a diagnostic (e.g., disease detection) and for monitoring (active surveillance or treatment monitoring).

Objectives: The primary objective was to determine the diagnostic accuracy of PROSTest (binary result: “yes” or “no”) using histopathological findings from the biopsy as the reference standard. The analysis was designed to model a clinical decision-making scenario in which PROSTest could inform the use of mpMRI and/or biopsy use—not to replace imaging, but to help identify patients who may safely defer further procedures under clinical supervision.

Clinical work-up and assessment: Patients were evaluated in accordance with the institutional protocol for primary prostate cancer detection. Most individuals presented with either an abnormal DRE or elevated PSA suggestive of PCa. No patient had received prior prostate-targeted therapy, undergone prostate surgery, or used oral 5α-reductase inhibitors before biopsy.

All patients underwent transrectal MRI–ultrasound fusion-guided biopsy using the UroNav System (Invivo Corporation, PHILIPS©, Gainesville, FL, USA). Targeted biopsy cores were obtained from all MRI-identified regions of interest under local anesthesia. Histopathologic evaluation was performed centrally, and tumors were graded according to the ISUP grade group (GG) system, in accordance with the ISUP 2014 consensus recommendations [16].

Blood collection: Following written, informed consent, a single peripheral blood sample was obtained from each participant prior to biopsy for PROSTest analysis. Samples were collected in proprietary RNA stabilization buffer tubes (Wren Laboratories, Branford CT, USA), in accordance with the standardized collection protocol [13,17]. Specimens were de-identified, assigned unique study codes, and shipped for analysis. Upon receipt, samples were stored at −80 °C and processed in batch at Wren Laboratories (CAP accreditation # 8640840). A concurrent blood sample was collected for standard-of-care PSA testing which was performed in the clinical laboratory using ELISA, according to established protocol.

PROSTest measurements: PROSTest was performed according to the previously described protocol [18]. Total RNA was isolated from whole blood using the Mini Blood Kit (Qiagen, Valencia, CA, USA). Complementary DNA was synthesized and real-time qPCR was conducted using pre-spotted TaqMan PCR plates (Life Technologies, Carlsbad, CA, USA) [13]. Expression levels were normalized to the reference genes *ALG9*, *TOX4* and *TPT1* and relative quantification was calculated using the ΔΔC_t_ method [13]. Results were reported as a PCa risk score ranging 0–100, which was dichotomized into positive and negative categories. A predefined cut-off score ≥ 50% indicated increased risk of PCa [13].

Statistical Considerations and Sample Size Calculation: The primary objective of this study was to assess the diagnostic performance of PROSTest using biopsy-confirmed cancer status (cancer versus no cancer) as the reference standard. Diagnostic accuracy metrics—including Sensitivity, Specificity, Positive Predictive Value (PPV), Negative Predictive Value (NPV)—were calculated. Each parameter was reported with two-sided 95% confidence intervals (CIs) derived using the Clopper–Pearson method. Test outcomes were classified as true positive (TP), false positive (FP), true negative (TN), or false negative (FN) based on concordance with biopsy findings. The pre-specified performance thresholds were an expected sensitivity of 80% and an expected specificity of 75% based on previous studies [19]. A power analysis was conducted (paired McNemar framework, alpha = 0.05, power = 80%, disease prevalence 80%—tertiary institution) and identifying 104 subjects would be sufficient. Receiver operating characteristic (ROC) curves were generated using continuous PROSTest scores to assess overall diagnostic performance. The area under the curve (AUC) and 95% CIs were reported as a measure of overall diagnostic accuracy.

Exploratory Clinical Utility Analysis (‘What-if’ Simulation): How can PROSTest reduce the MRI and/or biopsy burden by identifying patients who may not have cancer or require further molecular follow-up before image-guided biopsy? This calculation—the number of MRI and/or biopsies which could have been avoided if PROSTest was used—was conducted and its percents from an actual MRI and biopsy were also calculated.

Descriptive statistics were used for patient demographics. Continuous data are reported as median [interquartile range] or mean ± SD, as appropriate. Statistical analyses were performed using GraphPad PRISM (GraphPad Software, La Jolla, CA, USA, Version 11.0.0, www.graphpad.com, accessed on 12 January 2026) and MedCalc^®^ Statistical Software (MedCalc Software bvba, Ostend, Belgium, version 23.4.5, http://www.medcalc.org, accessed on 12 January 2026). A two-sided *p*-value *p* < 0.05 was considered statistically significant. No missing data were identified in the study dataset.

## 3. Results

**Figure 1:**
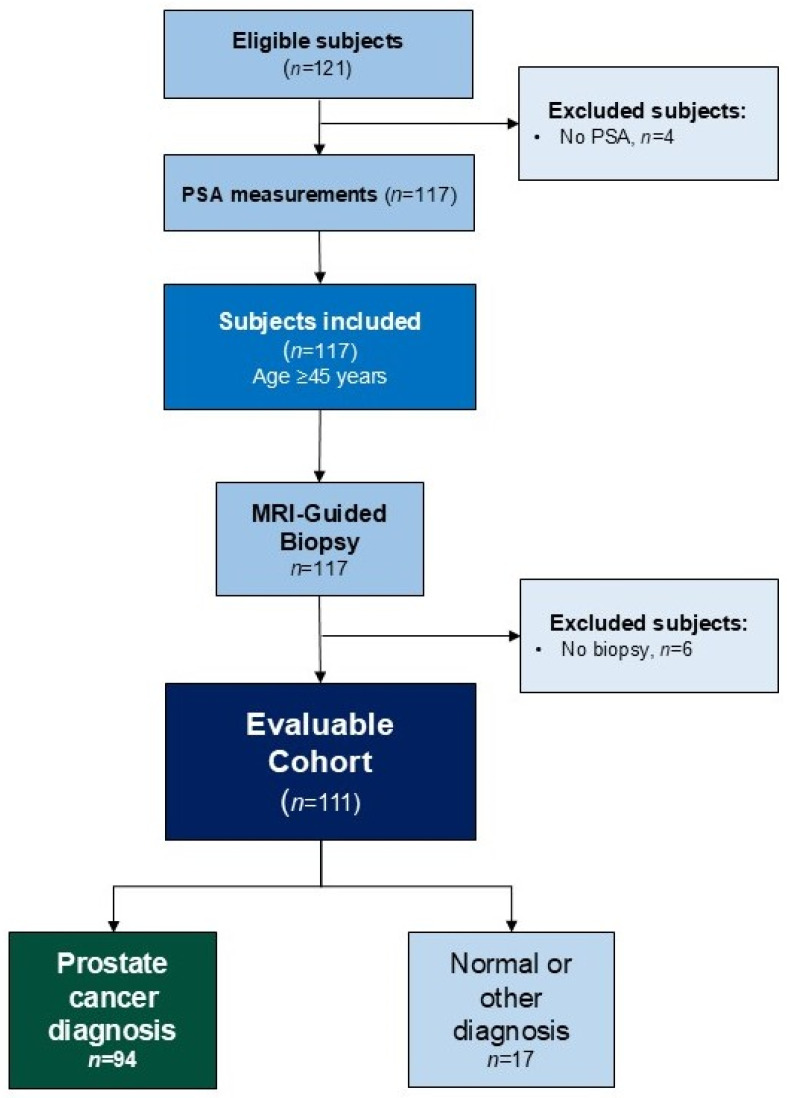
Overview of the study and outcomes (STARD diagram).

Of the 121 men assessed, 111 (91.7%) completed the full diagnostic assessment—including DRE, PSA, PROSTest and MRI with PI-RADS scoring—and subsequently underwent image-guided biopsy. The STARD diagram detailing patient selection and study inclusion is included in Figure 1.

The median age was 69 years (IQR: 63–74 years); the median PSA was 7.5 ng/mL (IQR: 5.8–11.4 ng/mL); 40 (36%) were DRE-positive and 104 of 111 (93.7%) had a PI-RADS score of 3–5.

PCa was diagnosed in 94 subjects (84.6%) while 17 men had no evidence of disease on biopsy. Eighty-six (91.5%) of the 94 with a biopsy-based diagnosis had clinically significant disease (csPCa; GG2-5). The demographics of the cohorts are included in Table 1.

An evaluation of the data demonstrated no significant differences in several factors (e.g., age, DRE+ve, number of biopsy cores or spectrum of PI-RADS score) although we did note subtle differences, e.g., GG3 and GG5 tended to have a higher PI-RADS score than those who did not have the disease. PSA also tended to be higher in GG4 and GG5 than in those with no disease. Of note, in the biopsy-negative cohort, four were DRE+ve and 70% had a PI-RADS score of 4–5.

We evaluated a novel molecular marker, PROSTest, for its utility in detecting disease. A pre-biopsy PROSTest was positive in 102/111 (91.9%) of subjects including 93/94 (98.9%) of those identified to have prostate cancer and 9/17 (53%) of men with no cancer (Chi^2^: 40.51, *p* < 0.0001). The AUROC was 0.95 ± 0.02 (95%CI: 0.89–0.98) (Supplementary Figure S2). The test exhibited an overall accuracy of 91% (84.1–95.6%) with a PPV of 91.2% (86.8–94.2%) and an NPV of 88.9% (51.6–98.4%). The sensitivity was 98.9% (94.2–100%) and the specificity was 47.1% (23.0–72.2%). The correlation between PROSTest scores, PI-RADS score and biopsy-detected disease is included in Table 2. Out of the nine patients with positive PROSTest but negative biopsy, six, one and two had PI-RADS scores 4, 3 and 2, respectively.

We next undertook a multivariate analysis (MVA) to identify the factors most associated with “predicting” disease detection (no disease vs. PCa; csPCa vs. GG1; no disease). The MVA demonstrated that both a PROSTest-positive score (≥50) and PI-RADS scores > 3 were significantly associated with PCa and csPCa detection (Table 3).

What if? Analysis: Given the concordance between PI-RADS scores and PROSTest, we evaluated multiple different scenarios to identify the best combination of factors for predicting who would have disease and whether this was a clinically significant disease at biopsy (Table 4). When applied in a hypothetical modeling scenario prior to imaging, PROSTest would have correctly identified eight of 17 men (47%) who were biopsy-negative. These findings suggest that PROSTest may help inform decisions to defer additional procedures in carefully selected, low-risk individuals under appropriate clinical follow-up.

## 4. Discussion

This prospective, single-center study in 111 men at risk for prostate cancer identified that a positive PROSTest demonstrated strong concordance with biopsy-proven PCa diagnosis across all Gleason grades and PI-RADS scores. Importantly, PROSTest functioned as a robust, independent predictor of PCa risk, maintaining diagnostic performance irrespective of imaging findings. PROSTest outperformed both DRE and an elevated (>3 ng/mL) PSA, highlighting its superior discriminatory capacity for clinically relevant disease. Additionally, it showed potential for helping to guide clinical decision-making based on imaging results. In this subgroup, PROSTest would have correctly identified nearly half of the men without cancer as lower-risk, suggesting a potential role in reducing unnecessary procedures when interpreted alongside clinical findings and mandatory safety overrides (e.g., markedly elevated PSA, abnormal DRE, or concerning MRI features). By providing readily accessible (blood test) molecular information about the tumor while maintaining 99% sensitivity for csPCa, PROSTest offers an efficient, non-invasive adjunct to PSA and MRI in the PCa diagnostic pathway.

MRI is now a standard approach for identifying men most at risk for PCa. Among patients with PSA ≥ 3 ng/mL, approximately 90% undergo an MRI that results in a PI-RADS score of 1–5 [20,21]. When focusing specifically on PI-RADS 3–5 findings—those typically triggering biopsy—the positivity rate ranges 55–80% [20,22,23,24]. The yield of csPCa among all MRI (PI-RADS 1–5) is relatively low (40–60% [20,21]), while the csPCa detection rate is approximately 52.0% in higher-risk groups [20,22,23,24]. In the current study, the majority of men (102/111–91.9%) had a PI-RADS of 4–5 score; 83 (81.4%) had a csPCA on biopsy. As importantly, of the nine men who had PI-RADS 1–3 scores, three had a csPCa. These data underscore the need for improved risk stratification prior to imaging to reduce unnecessary MRIs and avoid biopsies in men unlikely to have csPCa (see Table 2).

Wren Laboratories has developed the novel PROSTest (Supplementary Figure S1) [17,19,25,26,27,28,29,30,31,32,33,34,35,36,37,38,39,40,41,42] to address this unmet need by serving as a blood-based risk stratification tool that can precede mpMRI, thereby improving the specificity of the diagnostic pathway while maintaining safety. The test capability to preclude an unnecessary biopsy has been suggested in the prior clinical study [40]. In the current study, we evaluated the role of the assay prior to imaging.

In this study, PROSTest had an accuracy of 91% with a PPV of 91% and an NPV of 89% for detecting PCa. An evaluation of the PI-RADS 4–5 group identified that false-positive image-driven biopsies were undertaken in 11/73 (15%) with a PI-RADS 4 score and 1/29 (%) with a PI-RADS 5 score.

In a “what if” scenario, if PROSTest was used prior to MRI, the assay would have correctly identified six individuals who were biopsy-negative. This included the individual with a PI-RADS 5 (negative PROSTest, no cancer) and five of the six PI-RADS 4 men (all six negative, five had no evidence cancer). This would have decreased the need for biopsy in 6/102 (6%) of high-risk (by MRI) individuals. It is possible that a potential cancer was missed by biopsy in these cases. Our recommendation was a PROSTest follow-up and at least one more MRI-targeted biopsy after another mpMRI. For PI-RADS 3, we considered that the PROSTest could have value in helping mpMRI decision-making. A negative test result may obviate the need for immediate biopsy while a positive score could help adjudicate the need for a biopsy. Defining these exploratory findings would require a large, prospective study.

Nine individuals were PROSTest-positive but no cancer was detected. It is possible that the biopsy protocol missed cancers but this appears unlikely as ~13 cores were undertaken on average. Nevertheless, given the consistent association between PROSTest and disease, we recommend following these patients carefully as three also have a positive DRE and four have PSA levels > 9 ng/mL. Our recommendation would be a PROSTest follow-up and at least one more MRI-targeted biopsy after another mpMRI. Nine men had PI-RADS scores of 1–2 and underwent biopsy. PCa was identified in four; three of whom had csPCa. A positive PROSTest identified all four cancers, while a negative tested result would have identified two individuals (both PI-RADS score 1) as not having disease.

All but one (93/94) PCa was detected pre-biopsy by a positive PROSTest score; this included 85/86 (99%) csPCa. We recommend that all men with a positive PROSTest undergo image-guided biopsy. The one patient missed by the assay was a 58-year old male, DRE−ve, PSA of 8.16 ng/mL and a PI-RADS score of 4. The biopsy identified a GG3 lesion (Gleason score 4 + 3). Of note, a positive PROSTest also detected eight subjects who, on biopsy, were identified with GG1 lesions. Although often considered a clinically “benign” condition, GG1 particularly when associated with a positive DRE or a PSA > 10 ng/mL (two of eight in the current cohort) are treated more aggressively including radical prostatectomy and definitive local therapy. Understaging by biopsy cannot be ruled out in these cases and we therefore aim to have a follow-up with these patients after their next biopsy under active surveillance. PROSTest addresses the clinical and economic challenges associated with MRI capacity, patient anxiety, and overdiagnosis in PSA-screened populations. Additionally, in the nine/111 patients with high PSA, MRI-positive but biopsy-negative results, PROSTest may provide an additional molecular signal supporting closer follow-up or consideration of repeat biopsy.

In addition to clinical utility, cost is an important issue. Widespread use of mpMRI, despite its utility [43,44], is limited by cost, accessibility, and variability in interpretation, with up to 72% of U.S. urologists reporting limited access [45]. MRI is also expensive ($2500–$6000 in the U.S.), time-consuming, and not always available [46]. Additionally, resource requirements and time/travel commitments of at-risk subjects may limit its practicality, particularly for routine screening [47]. In principle, blood-based liquid biopsy tests are ~$1000. While an MRI is more clinically actionable (detection of disease), the cost–benefit of any blood test can be outweighed if the assay has a low sensitivity. PROSTest is highly sensitive (>95%) for detecting all PCa and is unlikely to generate additional economic burdens. PROSTest is NYSDOH and CAP-accredited and is currently available at Wren Laboratories (Connecticut, USA). The underlying laboratory techniques are standardized in routine clinical practice, facilitating scalable adoption and high-throughput implementation. Only a comprehensive economic analysis, however, will demonstrate the cost-effectiveness and value of this liquid biopsy test [48].

## 5. Conclusions

This prospective study shows that molecular evaluation of 27-marker genes in blood is at least as effective as an MRI for detecting prostate cancer in men before a diagnostic biopsy is undertaken. The assay identified >95% of csPCa and importantly helped to distinguish individuals at higher versus lower risk, providing additional information to guide biopsy decisions even among men with high PI-RADS (4–5) scores. The relatively small cohort size may limit external generalizability and contribute to wider confidence intervals around diagnostic performance estimates. Overall, PROSTest could be used as a complementary reflex test rather than a replacement for PSA. A potential implementation would be PSA-triggered PROSTest to refine pre-test probability and prioritize mpMRI and biopsy for higher-risk individuals, particularly in settings where MRI access is limited. Given that mpMRI is constrained by cost, limited accessibility, and substantial inter-reader variability, the blood assay may help resolve the problem of excessive imaging and unnecessary biopsy in men with elevated PSA. Larger multicenter validation cohorts will be necessary to confirm this.

## Figures and Tables

**Table 1:** Patient cohorts and demographics.

	PCa Cohort (Biopsy-Positive) (*n* = 94)					Non-PCa Cohort (Biopsy-Negative)	*p*-Value *
	GG1	GG2	GG3	GG4	GG5		
Number	8	44	32	3	7	17	N/A
Age	69 [49–77]	70 [47–81]	68.5 [55–83]	74 [73–78]	67 [65–72]	66 [57–82]	0.567
DRE +ve	0 (0%)	16 (36%)	15 (47%)	1 (33%)	4 (57%)	4 (24%)	0.767 **
PI-RADS	4 [2–4]	4 [2–5]	4 [4–5]	4 [4–4]	4 [4–5]	4 [1–5]	<0.0001 †
PI-RADS 1–2	1 (13%)	2 (5%)	0 (0%)	0 (0%)	0 (0%)	4 (24%)	0.13 **
PI-RADS 3	0 (0%)	1 (2%)	0 (0%)	0 (0%)	0 (0%)	1 (6%)	
PI-RADS 5	7 (87%)	41 (93%)	32 (100%)	3 (100%)	7 (100%)	12 (70%)	
Cores	13 [7–16]	13.5 [5–18]	14 [5–17]	13 [12–14]	13 [5–17]	14 [5–16]	0.945
No. of cores Biopsy-positive	1 [1–5]	6 [2–15]	7 [2–13]	6 [4–7]	11 [4–13]	0	0.0003
PSA (ng/mL)	7.8 [4.1–13.5]	5.9 [4.4–25.4]	5.7 [3–36.3]	15.6 [10.7–39.3]	12.3 [4.6–195]	5.4 [0.3–14.3]	0.0048 ††
PROSTest (+ve, %) ‡	8 (100%)	44 (100%)	31 (97%)	3 (100%)	7 (100%)	9 (53%)	N/A
PROSTest scores	83.3 [70.6–93.6]	83.6 [66.2–95.6]	83.2 [25–95.2]	88.4 [78–93.8]	85.2 [65.1–96.8]	74.8 [15.2–94.8]	N/A

Data are median [range] or as a % ( ). * Kruskal–Wallis test across all cohorts except ** Chi^2^ evaluation. † *p* = 0.001 Non-PCa vs. GG3. *p* < 0.0001 Non-PCa vs. GG5. †† *p* = 0.032 Non-PCa vs. GG4; *p* = 0.04 Non-PCa vs. GG5. ‡ This includes the % of subjects with a positive (≥50%) PROSTest score. N/A = not applicable, DRE = digital rectal examination, GG = ISUP Gleason Grade, and PCa = prostate cancer. The Non-PCa cohort includes all individuals who were negative.

**Table 2:** Relationship between PROSTest, MRI scores and, biopsy-detected cancer.

	PI-RADS 1 (*n* = 2)		PI-RADS 2 (*n* = 5)		PI-RADS 3 (*n* = 2)		PI-RADS 4 (*n* = 73)		PI-RADS 5 (*n* = 29)	
	Bx +ve (*n* = 0)	Bx −ve (*n* = 2)	Bx +ve (*n* = 3)	Bx −ve (*n* = 2)	Bx +ve (*n* = 1)	Bx −ve (*n* = 1)	Bx +ve (*n* = 62)	Bx −ve (*n* = 11)	Bx +ve (*n* = 28)	Bx −ve (*n* = 1)
PROSTest+ve	0	0	3	2	1	1	61	6	28	0
PROSTest−ve	0	2	0	0	0	0	1	5	0	1

Bx+ve = biopsy-positive, Bx−ve = biopsy-negative.

**Table 3:** MVA outputs for disease associations.

	Disease (Any PCa)			csPCa		
	Co-Efficient	t-Value	*p*-Value	Co-Efficient	t-Value	*p*-Value
DRE+ve	−0.113 ± 0.056	−0.232	0.82	0.114 ± 0.072	1.568	0.12
PI-RADS 4–5	0.323 ± 0.099	3.267	0.0015	0.351 ± 0.127	2.763	0.007
PROSTest+ve	0.746 ± 0.100	7.477	<0.0001	0.633 ± 0.127	4.971	<0.0001
PSA > 10 ng/mL	0.043 ± 0.068	0.635	0.527	0.001 ± 0.001	0.658	0.512

**Table 4:** PROSTest relationship with imaging and outcomes.

	PI-RADS Score	PCa	csPCA	Incorrect Results
PROSTest positive (pre-biopsy score ≥ 50)	1: *n* = 0	0	0	0
	2: *n* = 5	3 (60%)	2 (67%)	2 *
	3: *n* = 2	1 (50%)	1 (100%)	1 **
	4: *n* = 67	61 (91%)	54 (100%)	6 ***
	5: *n* = 28	28 (100%)	28 (100%)	0
		93/93 (100%)	85/85 (100%)	9 (9%)
PROSTest-negative (pre-biopsy score < 50)	1: *n* = 2	0	0	0
	2: *n* = 0	0	0	0
	3: *n* = 0	0	0	0
	4: *n* = 6	1 (17%)	1 (100%)	1
	5: *n* = 1	0	0	0
		0/1 (0%)	0/1 ^#^ (0%)	1/9 (11%)

* Both DRE−ve, PSA = 9 and 9.5 ng/mL. ** DRE−ve, PSA 1.3 ng/mL. *** 2 of 6 are DRE+ve, PSA 3.4–14.3 ng/mL (5/6 PSA < 10 ng/mL). ^#^ DRE−ve, PSA = 8.16 ng/mL.

## Data Availability

Due to privacy and ethical concerns, the data that support the findings of this study are not publicly available but are available on request from the corresponding author.

## Supplementary Materials

The following supporting information can be downloaded at https://www.mdpi.com/article/10.3390/cancers18050871/s1. Figure S1. Overview of PROSTest assay development. Figure S2. AUROC curve for the PROSTest. Table S1: PROSTest Marker Genes (*n* = 27).

## Abbreviations

The following abbreviations are used in this manuscript:

PCa	Prostate cancer
MRI	Magnetic resonance imaging
PI-RADS	Prostate Image Reporting and Data System
ML	Machine learning
RT-PCR	Reverse-transcription polymerase chain reaction
GG	Grade Group
NPV	Negative predictive value
PPV	Positive predictive value
IQR	Interquartile range
PSA	Prostate-specific antigen
DRE	Digital rectal examination
mpMRI	Multiparametric magnetic resonance imaging
HKG	Housekeeping genes
CTC	Circulating tumor cell
STARD	Standards for Reporting and Diagnostic Accuracy Studies
ELISA	Enzyme-linked Immunosorbent Assay
CI	Confidence interval
TP	True positive
FP	False positive
TN	True negative
FN	False negative
ROC	Receiver operating characteristic
AUC	Area under the curve
SD	Standard deviation
csPCa	Clinically significant prostate cancer
MVA	Multivariate analysis
