# Supplementary material for: Clinical Utility of PROSTest: A Prospective Study Suggesting Reduction in Unnecessary MRI and Biopsy in Men Evaluated for Prostate Cancer
Source: Cancers (Basel). 2026 Mar 8;18(5):871. doi: 10.3390/cancers18050871 (PMC12985235; doi:10.3390/cancers18050871)
Supplement: Supplementary file 1 [file cancers-18-00871-s001.zip › cancers-4175440-supplementary.pdf]

SUPPLEMENTARY INFORMATION:

Clinical Utility of PROSTest: A Prospective Study Suggesting Reduction in Unnecessary MRI and Biopsy in Men Evaluated for Prostate Cancer

Kambiz Rahbar[1,2], Martin Bögemann[2,3], Philipp Papavasilis[2,3], Abdel Halim[4], and Mark Kidd[4*]

1 Department of Nuclear Medicine, University Hospital Muenster, Muenster, Germany; Kambiz.Rahbar@ukmuenster.de
2 West German Cancer Centre Muenster, Germany; Martin.boegemann@ukmuenster.de
3 Department of Urology, University Hospital Muenster, Muenster, Germany; Philipp.papavasilis@ukmuenster.de
4 Wren Laboratories, Branford CT, USA; ahalim@wrenlaboratoratories.com; mkidd@wrenlaboratoratories.com

* Correspondence: mkidd@wrenlaboratoratories.com; Tel.: (+1)203 208-3464

Assay Development:

The assay was developed using a multi-step protocol (**Supplementary Figure S1**). These steps included firstly a signature derivation (5 tissue microarray datasets; mined using Random Forest classification algorithms and Gini importance values above the median value [1]). This detected 30 putative markers, 27 of which were retained (based on literature review). Secondly, biomarker specificity was assessed in thirty-two RNAseq datasets in The Cancer Genome Atlas (TCGA) project [2, 3]. These comprised TCGA-PRAD (PRAD, *n*=494: prostate cancers with exclusive adenocarcinoma histology, Gleason scores 5-10)) and 10.496 other solid tumors. Target gene expression was measured in blood samples and an algorithm was developed to score expression. This algorithm is scored 0-100 and is based on machine learning and includes an ensemble of 3 classifiers Random Forest (RF), Gradient Boosted Machines (GBM), and Support Vector Machines (SVM). These algorithms were selected due to their well-established theoretical foundations and a motivation to reduce the generalization error of the prediction [4]. Three classifiers were trained using *n*=433 samples (*n*=75 Controls, *n*=64 BPH, *n*=294 PCa) and model performance was evaluated using leave-one-out cross-validation. The PROSTest was evaluated in two independent testing sets (*n*=178; *n*=187) and a clinically relevant cut-off of 50 (on the 0-100) scale was identified [5]. This cut-off was examined in a prospective surgical

cohort (n=47) and has subsequently been evaluated in several clinical studies and validated [6-10].

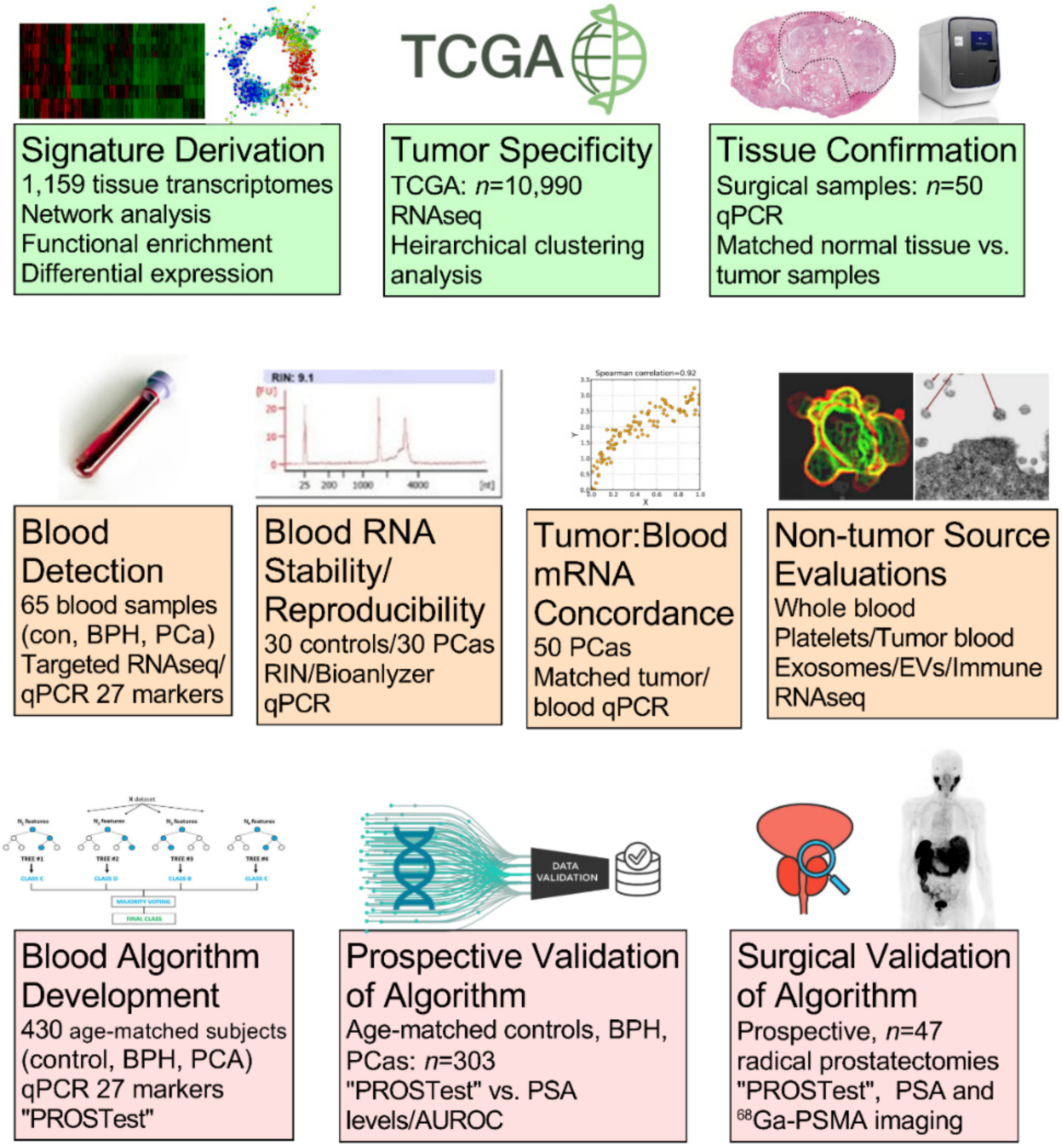

Supplementary Figure S1. Overview of PROSTest assay development.

Genes included in the PROSTest

The 27-marker genes are included in **Supplementary Table S1.**

Supplementary Table S1: PROSTest Marker Genes (*n*=27)

Symbol	Name	NCBI Chromosome Location	Principal Transcriptional Regulators*	Principal cell type specificity‡	Role in Prostate cancer† (Or other cancers)
AAMP	Angio-associated migratory cell protein	Chr.2: 218264129 - 218270209	AR	Non-specific	Yes<br>Highly expressed in PRAD<br>Negative prognostic<br>(Pan-cancer marker)
AR	Androgen receptor (Δ exon boundary – AR-2)	Chr.X: 67544032 - 67730619	AR	Prostatic glandular cells	Yes<br>Development, target, mutations
CHTOP	Chromatin Target Of PRMT1	Chr.1: 153633982 - 153646306	AR	Non-specific	Yes<br>(BRCA, GBM, urothelial, liver, melanoma)
EDC4	Enhancer Of MRNA Decapping 4	Chr.16: 67873023 - 67884514	AR	Gastric mucus, neuron, microglial, erythroid	Yes<br>(BRCA, LAML, OV)
FXYD7	FXYD Domain Containing Ion Transport Regulator	Chr.19: 35143250 - 35154302	AR	Neuron, NK- and T-cell, microglial cell	Not known<br>(COAD)
FYCO1	FYVE And Coiled-Coil Domain Containing 1	Chr.3: 45917899 - 45995824	AR	Cardiomyocyte Skeletal myocyte	Not known<br>(BLCA, renal)
HNRNPU	Heterogeneous Nuclear Ribonucleoprotein U	Chr.1: 244842123 - 244864720	AR	Monocyte (innate immune response)	Yes<br>Pan-cancer, prognostic<br>(Liver)
HPN	Hepsin	Chr.19: 35040506 - 35066573	AR	Hepatocyte	Yes<br>Diagnostic<br>(Renal)
KRT23	Keratin 23	Chr.17: 40922696 - 40937643	AR	Syncytio-trophoblast	Yes<br>Metastasis<br>(Urothelial)
MAN2B2	Mannosidase Alpha Class 2B Member 2	Chr.4: 6575174 - 6622403	AR	Macrophage (innate immune response)	Yes<br>(Endometrial)

MAX	MYC Associated Factor X	Chr.14: 65006101 - 65102695	AR	Platelet (hemostasis)	Yes<br>Prognostic, Proliferation<br>(Renal, thyroid)
MRPS25	Mitochondrial Ribosomal Protein S25	Chr.3: 15042251 - 15065337	AR	Late spermatid	Not known<br>(Renal)
NDUFS2	NADH: Ubiquinone Oxidoreductase Core Subunit S2	Chr.1: 161197377 - 161214395	AR	Non-specific	Yes<br>Risk factor<br>(Renal)
PPRC1	Peroxisome Proliferator-Activated Receptor Gamma, Coactivator-Related 1	Chr.10: 102132994 - 102150333	AR	Non-specific	Not known<br>(Renal)
RAD23A	RAD23 Homolog A, Nucleotide Excision Repair Protein	Chr.19: 12945814 - 12953643	AR	Erythroid cell	Not known
REPIN1	Replication Initiator 1	Chr.7: 150368228 - 150374044	AR	Intestinal goblet cell (mucin)	Yes<br>Proliferation<br>(PAAD, STAD, Renal Urothelial)
SDR39U1	Short Chain Dehydrogenase/Reductase Family 39U Member 1	Chr.14: 24439766 - 24442905	AR	Non-specific	Not known<br>(PAAD)
SETBP1	SET Binding Protein 1	Chr.18: 44680173 - 45068510	AR	Neuron, oligodendrocyte precursor	Yes<br>Driver gene<br>(Renal)
SLC14A1	Solute Carrier Family 14 Member 1 (Kidd Blood Group)	Chr.18: 45724123 - 45752520	AR	Astrocyte Adipocyte	Yes
SLC18A2	Solute Carrier Family 18 Member A2	Chr.10: 117241073 - 117279430	AR	Granulocyte	Yes<br>Prognostic
SMC4	Structural Maintenance of Chromosomes 4	Chr.3: 160399304 - 160434962	AR	Spermatocyte, Erythroid cell	Yes<br>Prognostic<br>(Renal, PAAD, liver, endometrial)
SPARC	Secreted Protein Acidic and Cysteine Rich	Chr.5: 151661096 - 151687054	AR	Connective tissue (fibroblast, stromal cell)	Yes<br>Progression, Metastasis<br>(Renal)
SQLE	Squalene Epoxidase	Chr.8: 124998478 - 125022283	AR	Hepatocyte	Yes<br>Prognostic<br>(PAAD, Lung, Renal, HNC)

STRIP1/ FAM40A	Striatin Interacting Protein 1	Chr.1: 110031577 - 110054641	AR	Late spermatid	Not known
STX12	Syntaxin 12	Chr.1: 27773183 - 27824452	AR	Adipocyte Endothelial cell	Not known
UNC45A	Unc-45 Myosin Chaperone A	Chr.15: 90929980 - 90954093	AR	Smooth muscle cell	Not known (renal, liver)
XPC	XPC Complex Subunit, DNA Damage Recognition and Repair Factor	Chr.3: 14145147 - 14178672	AR	Non-specific	Yes Risk (renal, urothelial)

*AR = androgen receptor signaling

‡Excluding female reproductive organs

†From the HPA (www.proteinatlas.org/) [11] and the GTEx Project (www.gtex.portal.org/home/gene) [12, 13] (Reviewed 9/2023)

NA = not annotated. No information identified for a role (PubMed search 9/2023).

BLCA = bladder cancer, BRCA = breast cancer, COAD = colon adenocarcinoma, GBM = glioblastoma, LAML = acute myeloid leukemia, OV = ovarian cancer, PRAD = prostate adenocarcinoma

An evaluation of the PROSTest is that it captures a Pan-PCa signature that is weighted towards Gleason 7-10 tumors. The biological pathways captured included gene expression related to DNA repair, homeostasis, metastasis, proliferation, mitochondrial function. Because PCa is a molecularly heterogeneous disease [2, 14, 15], we examined whether the 27-PROSTest gene signature was related to different genetics inputs e.g., known mutations, GWAS *etc*.

We examined the 27-gene signature against known commonly mutated genes in PCa [16]. Using a protein:protein interactomic approach, we identified an overlap in signaling with 12 (44%) of the PROSTest genes. In a second analysis, we evaluated the PROSTest against a panel of genes identified to be most frequently mutated in primary tumors [17, 18] and identified interactions with 14 (52%) of the PROSTest genes.

An evaluation of 269 known risk variants from GWAS databases [19] and genes that were within 100kb of these GWAS loci (total: n=309) identified that 18 PROSTest genes (67%) were connected with these GWAS loci.

In addition, genes commonly associated with hereditary cancer syndromes e.g., *BRCA1*, *BRCA2*, the 7 mismatch repair genes and *HOXB13* [20, 21] were also examined in the same fashion. Seven (26%) PROSTest genes were associated with these mutations.

The PROSTest gene signature therefore is significantly related to the molecular landscape and captures the transcriptional pathobiology of this heterogeneous tumor group.

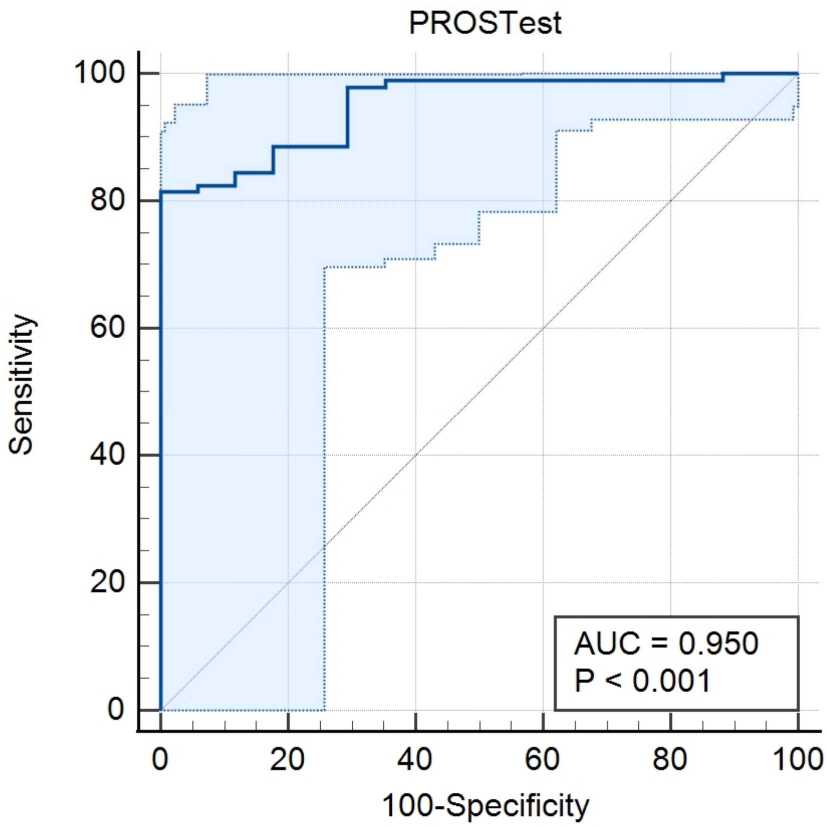

Supplementary Figure S2. AUROC curve for the PROSTest.

References

1. Breiman L. Random Forests. Machine Learning. 2001; 45: 5-32.
2. The Molecular Taxonomy of Primary Prostate Cancer. Cell. 2015; 163: 1011-25.
3. Johnston WL, Catton CN, Swallow CJ. Unbiased data mining identifies cell cycle transcripts that predict non-indolent Gleason score 7 prostate cancer. BMC Urol. 2019; 19: 4. doi: 10.1186/s12894-018-0433-5.
4. Maclin R, Opitz D. Popular Ensemble Methods: An Empirical Study. 2011. p. arXiv:1106.0257.
5. Modlin IM, Kidd M, Drozdov IA, Boegemann M, Bodei L, Kunikowska J, et al. Development of a multigenomic liquid biopsy (PROSTest) for prostate cancer in whole blood. Prostate. 2024; 84: 850-65.
6. Rogers C, SV K, Gulati A, Halim A. PROSTest, a Novel Liquid Biopsy Molecular Assay, Accurately Guides Prostate Cancer Biopsy Decision-Making in Men with Elevated PSA Irrespective of DRE Findings. Cancers. 2025; 17: 3908.

7. Rosin RD, Haynes A, Kidd M, Drozdov I, Modlin I, Halim A. Evaluation of a multigenomic liquid biopsy (PROSTest) for prostate cancer detection and follow-up in a Caribbean population. Cancer epidemiology. 2024; 92: 102642.
8. Kidd M, Rempega G, Kepinski M, Slomian S, Mlynarek K, Halim AB. Utility of the PROSTest, a Novel Blood-Based Molecular Assay, Versus PSA for Prostate Cancer Stratification and Detection of Disease. Prostate. 2026; 86: 307-13.
9. Rahbar K, Kidd M, Prasad V, David Rosin R, Drozdov I, Halim A. Clinical Sensitivity and Specificity of the PROSTest in an American Cohort. Prostate. 2025: e24858.
10. Rahbar K, Rosin RD, Kidd M, Halim AB, Sartor O. PROSTest, a Multigene Liquid Biopsy Signature, Effectively Stratifies Patients With High PSA for Prostate Biopsy. Prostate. 2026; 86: 43-52.
11. Karlsson M, Zhang C, Méar L, Zhong W, Digre A, Katona B, et al. A single-cell type transcriptomics map of human tissues. Science advances. 2021; 7.
12. The Genotype-Tissue Expression (GTEx) project. Nature genetics. 2013; 45: 580-5.
13. Enhancing GTEx by bridging the gaps between genotype, gene expression, and disease. Nat Genet. 2017; 49: 1664-70.
14. Wei L, Wang J, Lampert E, Schlanger S, DePriest AD, Hu Q, et al. Intratumoral and Intertumoral Genomic Heterogeneity of Multifocal Localized Prostate Cancer Impacts Molecular Classifications and Genomic Prognosticators. European urology. 2017; 71: 183-92.
15. Dawson NA, Zibelman M, Lindsay T, Feldman RA, Saul M, Gatalica Z, et al. An Emerging Landscape for Canonical and Actionable Molecular Alterations in Primary and Metastatic Prostate Cancer. Mol Cancer Ther. 2020; 19: 1373-82.
16. Barbieri CE, Baca SC, Lawrence MS, Demichelis F, Blattner M, Theurillat JP, et al. Exome sequencing identifies recurrent SPOP, FOXA1 and MED12 mutations in prostate cancer. Nat Genet. 2012; 44: 685-9.
17. Spans L, Clinckemalie L, Helsen C, Vanderschueren D, Boonen S, Lerut E, et al. The genomic landscape of prostate cancer. International journal of molecular sciences. 2013; 14: 10822-51.
18. Kumar A, Coleman I, Morrissey C, Zhang X, True LD, Gulati R, et al. Substantial interindividual and limited intraindividual genomic diversity among tumors from men with metastatic prostate cancer. Nat Med. 2016; 22: 369-78.
19. Conti DV, Darst BF, Moss LC, Saunders EJ, Sheng X, Chou A, et al. Trans-ancestry genome-wide association meta-analysis of prostate cancer identifies new susceptibility loci and informs genetic risk prediction. Nat Genet. 2021; 53: 65-75.
20. Giri VN, Beebe-Dimmer JL. Familial prostate cancer. Semin Oncol. 2016; 43: 560-5.
21. Russo J, Giri VN. Germline testing and genetic counselling in prostate cancer. Nature reviews Urology. 2022; 19: 331-43.
